# Comparison between epileptic seizure prediction and forecasting based on machine learning

**DOI:** 10.1038/s41598-024-56019-z

**Published:** 2024-03-07

**Authors:** Gonçalo Costa, César Teixeira, Mauro F. Pinto

**Affiliations:** Center for Informatics and Systems of the University of Coimbra, Department of Informatics Engineering, University of Coimbra, 3030-290 Coimbra, Portugal

**Keywords:** Biomedical engineering, Epilepsy, Biomedical engineering, Epilepsy

## Abstract

Epilepsy affects around 1% of the population worldwide. Anti-epileptic drugs are an excellent option for controlling seizure occurrence but do not work for around one-third of patients. Warning devices employing seizure prediction or forecasting algorithms could bring patients new-found comfort and quality of life. These algorithms would attempt to detect a seizure’s preictal period, a transitional moment between regular brain activity and the seizure, and relay this information to the user. Over the years, many seizure prediction studies using Electroencephalogram-based methodologies have been developed, triggering an alarm when detecting the preictal period. Recent studies have suggested a shift in view from prediction to forecasting. Seizure forecasting takes a probabilistic approach to the problem in question instead of the crisp approach of seizure prediction. In this field of study, the triggered alarm to symbolize the detection of a preictal period is substituted by a constant risk assessment analysis. The present work aims to explore methodologies capable of seizure forecasting and establish a comparison with seizure prediction results. Using 40 patients from the EPILEPSIAE database, we developed several patient-specific prediction and forecasting algorithms with different classifiers (a Logistic Regression, a 15 Support Vector Machines ensemble, and a 15 Shallow Neural Networks ensemble). Results show an increase of the seizure sensitivity in forecasting relative to prediction of up to 146% and in the number of patients that displayed an improvement over chance of up to 300%. These results suggest that a seizure forecasting methodology may be more suitable for seizure warning devices than a seizure prediction one.

## Introduction

Epilepsy is one of the most common neurological diseases. It affects around 1% of the world’s population and is characterized by recurrent seizures. One significant problem is the apparent unpredictable nature of seizures, especially in Drug-Resistant Epilepsy (DRE)^1^. Approximately one-third of patients with epilepsy suffer from DRE, a condition where the use of Anti-Epileptic Drugs (AEDs) is not enough to achieve seizure-free lives^2^. The inability to control seizures can lead to physical problems, such as an increased risk of accidental injury or even death, and psychological ones, such as neuropsychological deficits (memory loss and attention difficulties), depression, anxiety, or psychoses^3–5^. In cases where seizure control cannot be achieved through medication, surgery, or neurostimulation, the objective becomes informing the patients when a seizure will occur or estimating the seizure likelihood through warning devices^6^.

Researchers take two main approaches when developing an algorithm for a seizure warning device: seizure prediction and seizure forecasting. In seizure prediction, an alarm is raised when the algorithm detects the preictal period. This alarm-based view means that whenever an alarm is raised, the information that is given by the algorithm is that a seizure will occur in a well-defined period, the Seizure Occurrence Period (SOP), and only after a determined horizon that allows for intervention, the Seizure Prediction Horizon (SPH)^7^. From an optimal point of view, seizure prediction would be the ideal option. When a seizure is guaranteed to occur, it gives a warning, and actions can be taken accordingly. However, seizure generation is a very complex area, so as of today, seizure prediction is still linked to significant rates of false alarms, whether it is caused by underperforming algorithms or by cases where the brain is highly susceptible to a seizure but does not fully develop it^8^. These high rates of false alarms undermine entirely the objective of a warning device. Not only do they cause unnecessary stress initially, but as the false alarms keep happening, they cause alarm habituation and a consequent decrease in confidence in the system^9^.

On the other hand, seizure forecasting is much more flexible and feasible than seizure prediction. In forecasting, the algorithm does not warn a patient when a seizure is about to occur. Instead, it provides a constant analysis of the likelihood of a seizure. Similarly to what happens in weather forecasting, the algorithm should be able to detect low, moderate, and high-risk states and continuously relay the information to the user. Even though seizure forecasting takes a probabilistic approach, which is rarely fully confident of events, it avoids the crisp approach of prediction that, despite sometimes being correct, is linked to those damaging false alarms. Access to the probabilistic likelihood of seizures allows patients to make an informed decision based on a certain degree of uncertainty^9,10^.

The Electroencephalogram (EEG) is the most effective signal source for the diagnosis of epilepsy by outlining the epileptogenic part of the brain and analyzing the patient’s seizures. Analysis of the recorded EEG signals can be used to learn more about the features of epileptic seizures and allow for the distinction between its different phases^11^. The EEG of a patient with epilepsy can be segmented into distinct periods in time: the period before a seizure (preictal), the period during a seizure (ictal), the period after a seizure (postictal), and the period between the postictal and preictal periods of two consecutive seizures (interictal)^12^. The seizure prediction and forecasting algorithms aim to detect the brain patterns associated with the preictal period.

Our objective is to explore the effects of switching from a seizure prediction view to a forecasting one with the same data using similar patient-tailored Machine Learning algorithms. Our study made this comparison using a Logistic Regression, a voting system of 15 Support Vector Machines (SVMs), and a voting system of 15 Shallow Neural Networks (SNNs).

## Methods

**Figure 1:**
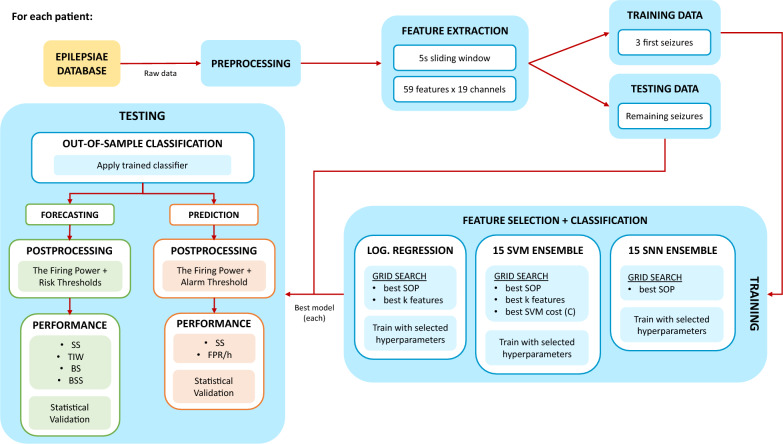
General overview of the study’s pipeline.

We developed six patient-specific methodologies with the goal of detecting the preictal period. The pipelines are grouped in pairs, each pair with a different classifier. In the postprocessing stage, these pairs diverge into two distinct branches: one for forecasting and one for prediction. To summarize, we developed three seizure forecasting models and three seizure prediction ones. Figure 1 presents an overview of the employed pipeline.

### Data

This study used a subset of 40 patients with Temporal Lobe Epilepsy (TLE), the most common type of focal epilepsy (17 females and 23 males, with a mean age of 41.43±15.87 years) from the European Database on Epilepsy (EPILEPSIAE). For more detailed information about the selected patients, please refer to Supplementary Table S1 of the supplementary materials. The data was collected from DRE patients during presurgical monitoring at the Universitätsklinikum Freiburg in Germany and contains 224 seizures. We only included patients who had experienced at least four lead seizures, with a minimum separation of 4.5 hours between each seizure. The data was collected using scalp EEG with a sampling rate of 256 Hz and 19 electrodes placed following the International 10-20 System. The Ethical Committees of the three hospitals involved in the EPILEPSIAE database approved the use of this data for research purposes (Ethik-Kommission der Albert-Ludwigs-Universität, Freiburg; Comité consultatif sur le traitement de l’information en matière de recherche dans le domaine de la santé, Pitié- Salpêtrière University Hospital; and Ethics Committee of the Centro Hospitalar e Universitário de Coimbra). All studies were carried out in accordance with the necessary guidelines and regulations, and all subjects and/or their legal guardian(s) provided informed written consent.

### Preprocessing

We preprocessed the raw EEG data using a Convolutional Neural Network (CNN)-based artifact removal model proposed by Lopes et al.^13^. This algorithm automatically eliminates artifacts from EEG signals, including eye blinks, eye movements, muscular activity, heart activity, and electrode connection interference, achieving results comparable to those of experts.

With EEG recordings from the EPILEPSIAE database, including data used in the present work, the model was trained on raw and manually preprocessed EEG segments to replicate the experts’ actions during data preprocessing. The experimental findings demonstrated that the proposed model effectively reduced the impact of EEG signal artifacts without human intervention, making it well-suited for long-term real-time scenarios.

### Feature extraction

Following the preprocessing phase, we divided the EEG signals into non-overlapping 5-second windows to extract relevant features. The choice of a 5-second window aligns with current practices in seizure prediction and forecasting^14–18^. However, there are other window length options, such as using epoch reduction to obtain a more responsive algorithm^19^.

We extracted univariate linear features as these features require relatively lower computational power than multivariate features and are more well-understood than nonlinear ones^14,15,18,20–24^.

**Figure 2:**
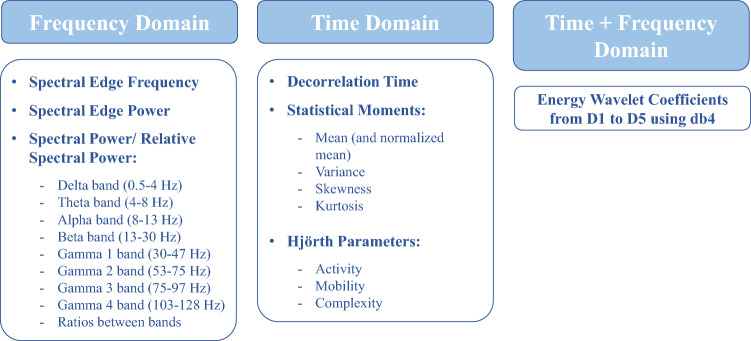
Summary of the univariate linear features used in this work.

Consequently, we extracted 59 univariate linear features on each of the 19 EEG channels in each 5-second window. Concerning the frequency domain, we extracted the relative spectral power of bands delta (0.5-4 Hz), theta (4-8 Hz), alpha (8-13 Hz), beta (13-30 Hz), and four gamma sub-bands: gamma band 1 (30-47 Hz), gamma band 2 (53-75 Hz), gamma band 3 (75-97 Hz), and gamma band 4 (103-128 Hz), the ratio between the bands, spectral edge frequency and power at 50%. Regarding the time domain, we computed the four statistical moments (mean, variance, skewness, kurtosis), Hjörth parameters (activity, mobility, complexity), and decorrelation time. Additionally, we extracted time-frequency features, namely the energy of five wavelet detail coefficients (from D1 to D5, using the db4 mother wavelet). Figure 2 provides a summary of the features we used. For a detailed description of these features, please refer to the Supplementary Information.

### Training and testing sets

This methodology is patient-specific, meaning that the classifiers are trained using data from one patient and tested on different data from that same patient. There is also an optimization of several hyperparameters using the training data for each patient. The process is then repeated for all patients. So, we divided each patient’s set of seizures into a group for training and another for testing. We used the training set, composed of the first three seizures per patient, to determine different optimal hyperparameters (the SOP duration for every classifier, the number of features for the Logistic Regression and the SVM ensemble, and the cost hyperparameter (*C*) for the SVM ensemble) and to train the classifier. Then, we applied the trained model to the testing set, composed of the remaining seizures per patient, and used it to evaluate the classifier’s performance. Consequently, we used 120 seizures in the training phase across all patients, corresponding to 3889 hours (4.05 ± 2.58 days per patient) of data, and 104 seizures in the testing phase, corresponding to 1595 hours (1.66 ± 1.24 days per patient).

### Classifier

**Figure 3:**
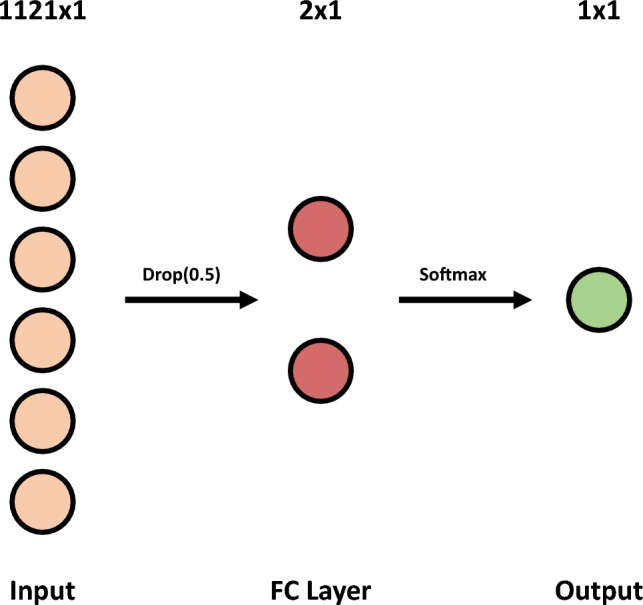
Shallow Neural Network architecture based on Lopes et al.^21^. It is composed of an input layer (Input), a dropout layer with a 50% rate (Drop(0.5)), a fully connected layer (FC layer), and an output layer (Output).

We used three approaches for the classification stage. The most straightforward approach has a Logistic Regression classifier with the class weights considered. For the following approach, we used a voting system of 15 SVMs with a linear kernel and optimization of the *C* hyperparameter. Finally, the last approach used a voting system of 15 SNNs with the architecture presented in Fig. 3, an Adam optimizer with a 3e-4 learning rate, and a binary cross-entropy loss function^21^. We set the training to 500 epochs, and to avoid overfitting, we considered early stopping regularization with a 50-epoch patience. Other possible classification options popular in the state-of-the-art include decision trees, decision forests, k-Nearest Neighbors, and deep learning approaches^14,21,25–28^.

### Training methodologies

We divided the samples into two distinct classes: preictal (level 1) and interictal (level 0). The preictal class contains the SPH; the remaining preictal length corresponds to the SOP. The SPH duration was set to 10 minutes as it is the most suitable time interval for rescue medication intake^29^. Regarding the SOP, we analyzed seven options, ranging from 20 to 50 minutes with 5-minute intervals. Higher values would result in a preictal period longer than one hour, which may not be practical for clinical usage^30^. We also standardized the training set features with the *z*-score normalization method.

Due to the relative rarity of seizures, the amount of interictal samples is considerably more significant than preictal ones, with the ratio of preictal to interictal samples on the order of magnitude of $10^{-2}$. To fix this imbalance, we implemented a class balancing step. There exist several approaches in the state-of-the-art to deal with this problem^31^. For the Logistic Regression model, we dealt with this step by training the classifier taking into consideration class weights^14^. As interictal samples are much more frequent than preictal samples, the weight for the interictal class will be smaller. This relationship is inversely proportional. The class weights are given by:

1 $$\begin{aligned} w_{i}=\frac{N_{Total \ Samples}}{2\cdot N_{i \ Samples}}, i = 0, 1. \end{aligned}$$

where $N_{Total \ Samples}$ is the total number of samples, $N_{i \ Samples}$ is the number of samples of class *i*, class 0 corresponds to interictal, and class 1 corresponds to preictal.

**Figure 4:**
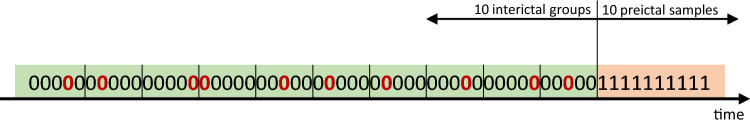
Random undersampling of the interictal class on a hypothetical seizure with ten preictal samples. The red-colored samples represent the randomly selected samples from each group.

For the SVM and SNN ensembles, we performed class balancing through a systematic random undersampling^14,15,21,23^. This procedure results in an equal number of interictal and preictal samples while sampling more or less equally spaced samples from the most represented class (the interictal class), allowing it to cover approximately the full temporality of the signal. It does so by dividing the interictal class into *n* groups, where *n* is the number of preictal samples, and randomly selects a sample from each group. Maintaining samples from every interictal interval guarantees higher representativeness. Figure 4 illustrates the random undersampling technique.

Before training the classifier, we performed feature selection to find the most discriminative features. We also analyzed seven values for the *k* number of most discriminative features (3, 5, 7, 10, 15, 20, 30) and implemented the ANOVA (Analysis of Variance) *f*-test for the Logistic Regression and a Random Forest classifier for the SVM ensemble. We did not perform feature selection on the SNN ensemble, allowing the SNN to determine the most discriminative features for the problem at hand.

To determine the optimal SOP, *k* number of features, and the SVM *C* hyperparameter ($2^{-10}$, $2^{-8}$, $2^{-6}$, $2^{-4}$, $2^{-2}$, $2^0$, $2^2$, $2^4$, $2^6$, or $2^8$), we adopted a grid-search strategy with a Leave-One-Out Cross-Validation (LOOCV). The grid-search results for each patient can be found in Supplementary Tables S2, S3, and S4 of the supplementary materials. This method uses 3-fold cross-validation, using two seizures to train and one for validation from the training set. To evaluate the model, we first calculated the confusion matrix based on the validation results of each combination. Then, using the Sample Sensitivity ($SS_{Sample}$) and Sample Specificity ($SP_{Sample}$), we calculated the geometric mean, a performance metric capable of revealing the trade-off between these two values: $\sqrt{SS_{Sample} \cdot SP_{Sample}}$. After assessing every combination, we selected the one with the highest geometric mean.

Finally, using the previously determined optimal hyperparameters, we trained the classifiers. As for the SVM and SNN ensembles, we executed the procedure 15 times for each patient. This procedure resulted in 15 models and 15 outputs per sample, so we used a voting system strategy where the most predominant class determined the final output.

### Postprocessing

After classification, we performed a regularization step to reduce the noise and smooth the output.

We applied the Firing Power method proposed by Teixeira et al.^32^. It uses a sliding window analysis to determine the preictal sample ratio. The mathematical formula for this method is:

2 $$\begin{aligned} fp\left[ n \right] = \frac{\sum _{k=n-\tau }^{n}O\left[ k \right] }{\tau }, \end{aligned}$$

where *fp*[*n*] is the “Firing Power,” $\tau$ is the number of samples in the selected window, and $O\left[ k \right]$ is the model’s output. Essentially, this method involves applying a moving average low-pass filter to the binary output of the classifiers, resulting in a continuous probability of a sample being preictal.

The next step is what differentiates the prediction and forecasting methodologies. For the prediction approach, the model raises an alarm once the Firing Power surpasses a certain threshold. We considered a conservative value of 0.7 for the threshold^14,15,23^ and defined a refractory period equal to the preictal duration, where no other alarms can be raised. For the forecasting approach, the model considers a high risk of seizure if the Firing Power surpasses a value of 0.7, moderate risk if it surpasses 0.3, and low risk if it is 0.3 or less.

### Performance evaluation

To evaluate the performance of the prediction and forecasting models, we used the standard metrics for each approach. Some of these metrics vary between approaches.

#### Prediction

For the seizure prediction pipelines, we computed the Seizure Sensitivity (SS) and the False Positive Rate per Hour (FPR/h)^7^.

The SS is the ratio between the number of predicted seizures, represented by the number of true alarms, and the total number of seizures, as described by:

3 $$\begin{aligned} SS=\frac{Number \ of \ predicted \ seizures}{{Total \ number \ of \ seizures}}=\frac{Number \ of \ true \ alarms}{{Total \ number \ of \ seizures}}. \end{aligned}$$

The FPR/h (Eq. 4) represents the number of alarms that do not correspond to seizures raised in one hour. It is defined by the ratio between the number of false alarms and the interval during which the model can raise one - the interictal period without the refractory periods.

4 $$\begin{aligned} FPR/h=\frac{Number \ of \ false \ alarms}{Interictal \ duration - Number \ of \ false \ alarms * (Refractory \ period)}. \end{aligned}$$

Regarding statistical validation, we used a surrogate time series analysis to determine if the developed algorithm performed above chance level with statistical significance. In this procedure, we randomly placed the original onset time of each seizure within the interictal period. We then used the resulting surrogate seizure times to calculate the algorithm’s SS. This process was repeated 30 times, and the average SS obtained from the surrogate analysis was compared to the SS achieved by the proposed methodology. The algorithm performed better than chance if the SS was higher than the surrogate one with statistical significance, considering a significance level of 0.05. To quantify the degree of statistical validation, we calculated the Improvement over Chance (IoC). This metric depicts the ratio between the number of statistically validated patients and the total number of patients.

#### Forecasting

For the seizure forecasting pipelines, we computed the SS, the Time in Warning (TiW), the Brier Score (BS), and the Brier Skill Score (BSS)^9,10,33–35^.

The SS is the ratio between the number of forecast seizures, which account for the number of seizures where the algorithm outputs a high-risk state in the SOP, and the total number of seizures (Eq. 5).

5 $$\begin{aligned} SS=\frac{Number \ of \ forecast \ seizures}{{Total \ number \ of \ seizures}}. \end{aligned}$$

The TiW is the fraction of time spent in a warning. More specifically, it is the time the model spends in a high-risk state divided by the total time.

The BS evaluates the performance of probabilistic forecasting methods by measuring the difference between these continuous forecasts and the observed seizure rates. It assesses the degree of success in matching different forecast probabilities to its observed probabilities of suffering from a seizure. For good forecasts, the BS tends to 0, while for bad forecasts, it tends to 1. Essentially, as Eq. (6) shows, the BS is the mean squared error between the forecast and the observation:

6 $$\begin{aligned} BS=\frac{1}{N} \sum _{i=1}^{N} (f_i-o_i)^2, \end{aligned}$$

where $f_i$ is the forecast probability, $o_i$ is the observation, and *N* is the number of forecast time points.

The BSS is defined as the improvement of the BS over an uninformed reference forecast $BS_{ref}$:

7 $$\begin{aligned} BSS=1-\frac{BS}{BS_{ref}}. \end{aligned}$$

If the BSS approximates 1, the forecasts are successful and can truly show the probability of seizures. If the forecasts are not better than the uninformed reference, the BSS will tend to 0. If the forecasts are worse than the reference, the BSS will be negative.

For the $BS_{ref}$, we used a randomly shuffled forecast. These surrogate forecasts randomly draw probabilities from the same distribution as the classifier’s output. This process was repeated 1000 times, and the $BS_{ref}$ was calculated by the mean of the BS from all surrogates^9,35,36^.

Concerning statistical validation and IoC, we used two surrogate time series analyses of different metrics. The first one was the same as for prediction. The second one checked if the BS obtained by the model was better than chance. For this, we used the $BS_{ref}$ value, obtained through the mean of the BSs of 1000 surrogate forecasts, and compared it to the proposed model’s BS. The same one-sample *t*-test was performed. The algorithm performed better than chance if the obtained BS is higher than the surrogate and statistically relevant.

## Results and discussion

Table 1 presents the overall seizure prediction and forecasting results for every developed methodology. Please refer to Supplementary Tables S2, S3, and S4 of the supplementary material for each patient’s results using the three classifiers.

**Table 1:** Overall results for the three Machine Learning pipelines.

Model	Prediction			Forecasting					
	SS	FPR/h	IoC SS	SS	TiW	BS	BSS	IoC SS	IoC BS
Logistic regression	0.13±0.26	0.36±0.40	12.5% (5 in 40)	0.28±0.37	0.13±0.14	0.19±0.11	0.01±0.15	37.5% (15 in 40)	50% (20 in 40)
15 SVMs ensemble	0.13±0.21	0.73±0.85	17.5% (7 in 40)	0.32±0.37	0.19±0.17	0.23±0.13	-0.01±0.14	32.5% (13 in 40)	47.5% (19 in 40)
15 SNNs ensemble	0.12±0.20	0.48±0.66	20% (8 in 40)	0.22±0.33	0.13±0.14	0.18±0.11	-0.04±0.18	25% (10 in 40)	45% (18 in 40)

### Seizure prediction

Regarding the prediction results, we can see that the SS for all classifiers is almost the same, at approximately 0.13. The FPR/h did not follow the same trend, with the Logistic Regression having the best value and the SVM ensemble the worst. These lower SS values are expected as the algorithm uses a 0.7 Firing Power threshold, taking a more conservative approach. However, the poor values for the FPR/h raise concerns about the practical applicability of the system in real-life scenarios^7^. Results for the IoC were also poor for every model, with only a three-patient difference from the worst to the best classifier.

We can make an interesting comparison between the Logistic Regression and SVM ensemble models. They both produce the same mean SS, but there is a 103% increase in the FPR/h from the Logistic Regression to the SVM ensemble. This disparity, added to the fact that the Logistic Regression is far computationally lighter than the 15 SVM ensemble (average of 7.34 seconds vs. 196.18 seconds per patient, respectively), suggests that the former may be a better option.

### Seizure forecasting

Regarding the forecasting results, the SS values differed between models, with the best belonging to the SVM ensemble. However, this model also obtained the highest TiW and BS. On the other hand, the Logistic Regression obtained an SS close to the SVM model and the lowest TiW and BS. Additionally, it is the only model with a positive BSS, meaning its performance is better than an uninformed forecast.

Concerning the statistical validation, the SS IoC obtained a five-patient difference between models, and the BS IoC obtained a two-patient difference. Furthermore, the IoC results for the BS were up to 80% better than the SS ones. This increase is because the BS is the mean squared error of the forecasts relative to the observations. So, while statistical validation using the SS only assesses the classifier’s ability to classify the preictal state as high-risk, the one using the BS also considers correct identification of the interictal period with low seizure probabilities. This way, it can validate patients based on the model’s aptness to correctly identify interictal samples as low-risk states, even if they cannot forecast the seizure. Furthermore, the BS is a more independent metric than the SS, as the latter is entirely dependent on the determination of a threshold, and the former only deals with the probabilistic output.

### Comparison with the state-of-the-art

We will use the Logistic Regression results to compare to the state-of-the-art. For prediction, it is the model with the best SS and FPR/h trade-off. For forecasting, despite not having the best SS, it has the best TiW, BS, BSS, and largest IoC.

Table 2 displays the performance results for several studies in the state-of-the-art and our Logistic Regression prediction model.

**Table 2:** Seizure prediction performance for studies under comparison.

Study	No. of patients	SS	FPR/h	IoC
Lopes et al. (2023)^21^	41	0.34	0.90	51%
Pinto et al. (2022)^15^	93	0.16	0.21	32%
Pinto et al. (2021)^23^	19	0.37	0.79	32%
Alvarado-Rojas et al. (2014)^37^	53	0.47	0.94	13%
Developed methodology	40	0.13	0.36	12.5%

These studies used the EPILEPSIAE database and implemented statistical validation. Except for Lopes et al.^21^, they also took the same approach as the present work regarding determining the SOP by testing a range of values.

Compared to Lopes et al.^21^ and Alvarado-Rojas et al.^37^, our model has the lowest SS. However, we achieved better FPR/h results than these studies. The choice of the alarm threshold value may partly explain the worse SS and better FPR/h values. Both selected studies use 0.5 as an alarm threshold, while we take a more conservative approach, choosing a threshold of 0.7. Two papers by Pinto et al.^15,23^ also used a 0.7 alarm threshold. The first study^15^ achieved comparable results. The second study^23^ obtained a higher SS, but only at the cost of a much higher FPR/h. The statistical validation in the form of the IoC we achieved is overall the lowest, closely followed by Alvarado-Rojas et al.^37^.

Table 3 displays the performance results for several studies in the state-of-the-art and our Logistic Regression forecasting model.

**Table 3:** Seizure forecasting performance for studies under comparison.

Study	Database	No. of patients	SS	TiW	BSS	IoC
Viana et al. (2022)^27^	ZUH KCL’s clinical trial	6	0.74	0.31	–	83%
Pal Attia et al. (2022)^28^	ZUH KCL’s clinical trial	6	0.69	0.37	–	67%
Proix et al. (2021)^36^	NeuroPace	18	–	–	0.23	83%
Nejedly et al. (2019)^34^	NeuroVista Canines	4 (dogs)	0.79	0.18	–	100%
Karoly et al. (2017)^35^	NeuroVista	9	0.55	0.25	0.05	100%
Cook et al. (2013)^26^	NeuroVista	10	0.61	0.23	–	90%
Developed methodology	EPILEPSIAE	40	0.28	0.13	0.01	50%

Our SS was again the worst among the group. On the other hand, the TiW was the lowest. We achieved a BSS close to Karoly et al.^35^ but much lower than Proix et al.^36^. Our IoC is also the lowest among the selected studies. However, it is noteworthy that this percentage corresponds to a total of 20 validated patients. As a comparison, most selected studies have a sample size of 10 patients or less.

The poor results may be caused by the database we used. All selected studies use ultra-long-term databases of invasive or sub-scalp EEG, with data of up to months of regular patient activity. On the other hand, the EPILEPSIAE database contains, at most, a few days of data from presurgical monitoring, a context designed to force seizure occurrence, and recorded with scalp EEG, a much more noisy data source. As the days in these conditions pass and the effects of the medication leave the patients’ systems, the brain function changes over time, and the number of seizures increases. Ultra-long-term databases with abundant data and of better quality, as they contain brain activity associated with regular daily life, would be better suited for seizure forecasting studies.

### Seizure prediction vs. seizure forecasting

However, the main goal of this study is to determine if a seizure forecasting approach produces better and more optimistic results than seizure prediction. The way to address this challenge is to compare the SS values. We can see a significant improvement in the SS in all three models when moving from prediction to forecasting. Based on the results in Table 1, there is a 115% increase for the Logistic Regression, 146% for the SVM ensemble, and 83% for the SNN ensemble. This trend also exists for statistical validation, showing an IoC increase of 300%, 171%, and 125%, respectively.

To better understand and visualize the model’s different dynamics and the differences in performance, we produced and analyzed Firing Power plots that show the Firing Power curve over time. As described in the Postprocessing section, the Firing Power curve is the result of applying Eq. (2) to the binary output of the classifiers. This technique gives us a smooth output that, essentially, corresponds to the probability of any given moment being preictal. In light brown, the figures also show the true SOP (corresponding to the total preictal duration without the 10-minute SPH). For the prediction output, the figure marks the moment an alarm is raised (once it crosses the 0.7 threshold) with a red triangle. For the forecasting output, the figure is color-coded to show which risk state the algorithm detects (green for low, yellow for moderate, and red for high).

**Figure 5:**
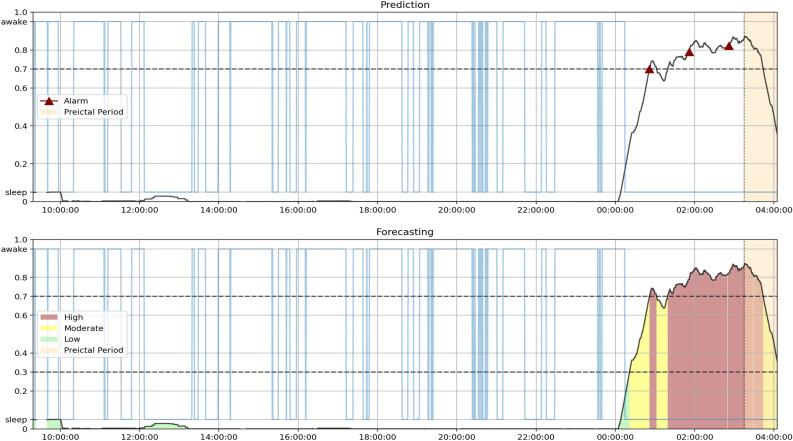
Plot of the Firing power curve with the alarms (seizure prediction) and with the risk zones (seizure forecasting) for seizure 8 of patient 30802 using the Logistic Regression model. The light blue curve represents the sleep/awake state determined by the algorithm described in Ref.^38^.

Firstly, the distinction between correctly predicted and forecast seizures in the SS can explain the performance increase. In practical terms, there is a reason why a forecasting algorithm can have a better SS than a prediction one. Figure 5 contrasts the Firing Power for seizure 8 of patient 30802 using the Logistic Regression model. The prediction plot shows that once the Firing Power curve crosses the threshold, it stays above it. So, to avoid constant alarms, when one is raised, it is associated with a refractory period, usually the duration of the SPH and SOP combined. In this case, the algorithm raised an alarm right before the SOP. Therefore, even though the output was above 0.7, due to the refractory period and the crisp nature of seizure prediction, the algorithm did not raise an alarm during the SOP and failed to warn the patient in the designated period. On the other hand, the forecasting model was in a high-risk state during part of the SOP. Accordingly, for this approach, the model was successful.

**Figure 6:**
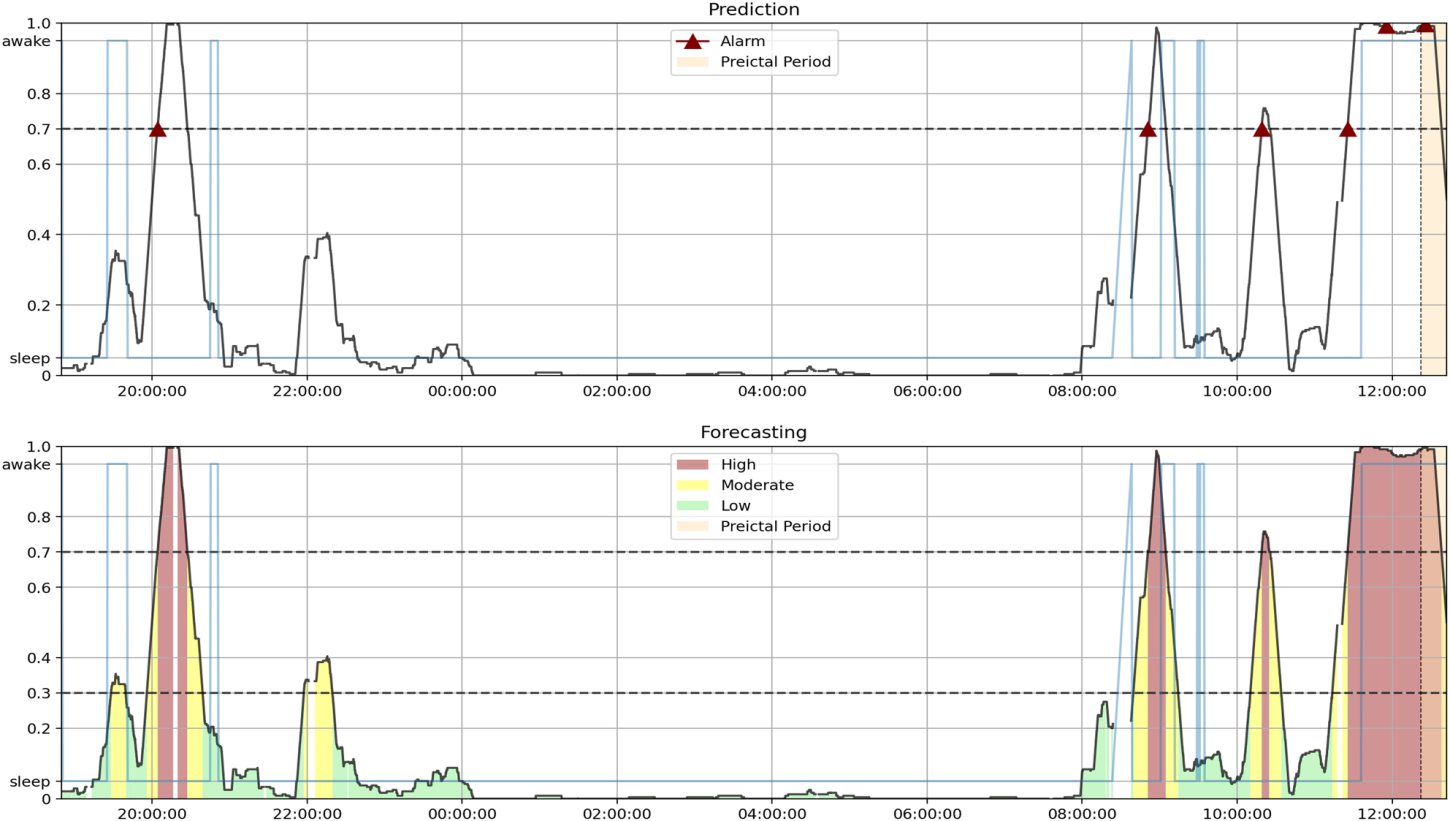
Plot of the Firing Power curve with the alarms (seizure prediction) and with the risk zones (seizure forecasting) for seizure 6 of patient 114902 using the Support Vector Machine model. The light blue curve represents the sleep/awake state determined by the algorithm described in Ref.^38^.

Seizure prediction algorithms also fail when dealing with potential seizure self-termination brain mechanisms. There are times when a seizure may be about to occur, but the brain’s natural self-regulation mechanisms may stop it before it fully develops. These mechanisms may be somewhat impaired in individuals with epilepsy, allowing for some seizures to occur. However, they still work to a certain degree. Seizure self-termination may also occur in seizures that have not fully developed yet^8^. Figure 6 shows the SVM ensemble output of patient 114902’s 6th seizure. There is a clear elevation in the Firing Power curve slightly after 20:00:00, and two between around 09:00:00 and 11:00:00. These elevations may be caused by a seizure close to occurring that was terminated by these specific brain dynamics. If that is the case, the probabilistic approach of forecasting can avoid these occasions where the model is wrong.

**Figure 7:**
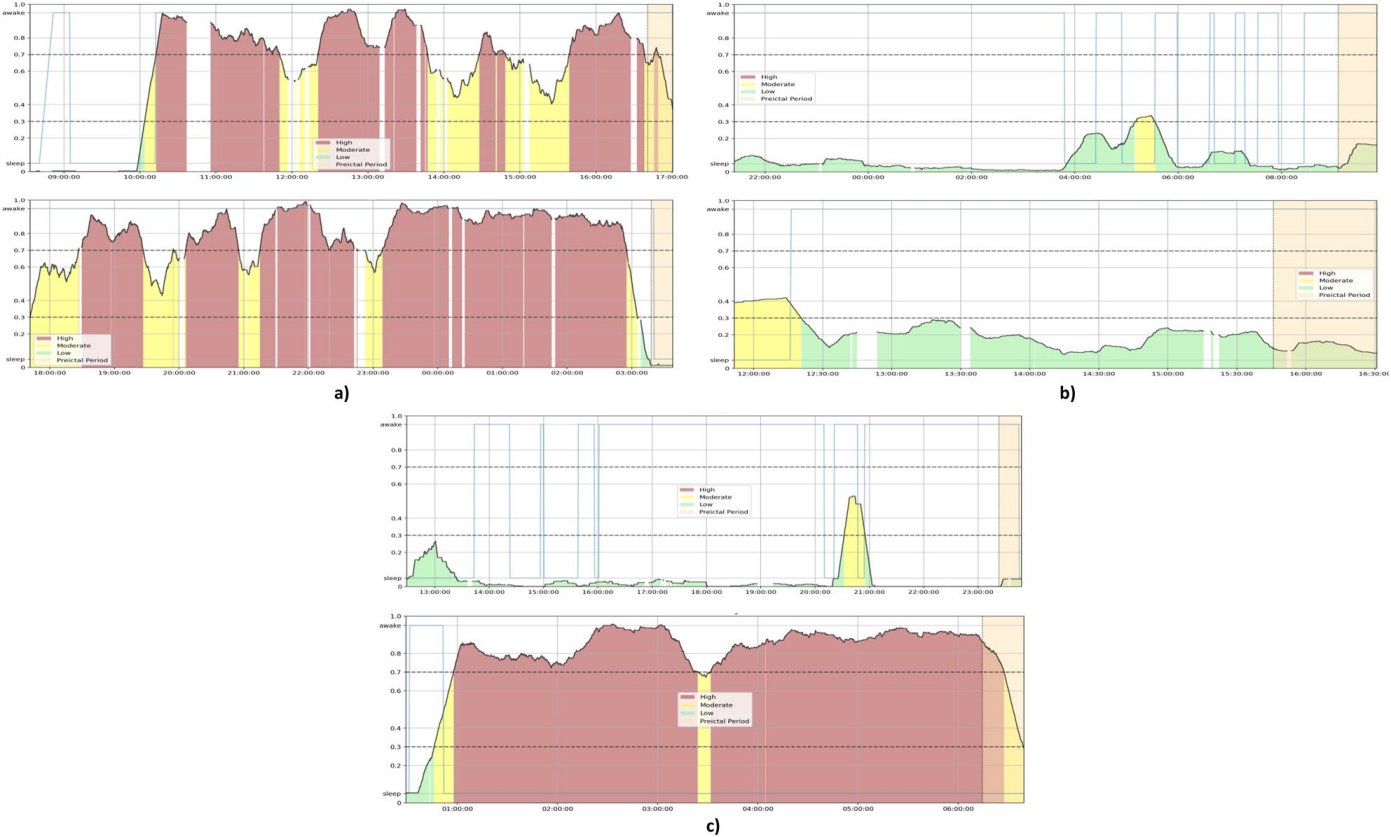
Plot of the Firing Power curve with the risk zones using the Shallow Neural Network model for (**a**) seizures 5 (top) and 6 (bottom) of patient 94402 (** b**) seizures 4 (top) and 5 (bottom) of patient 58602 (**c**) seizures 4 (top) and 5 (bottom) of patient 30802. The light blue curve represents the sleep/awake state determined by the algorithm described in Ref.^38^.

Nevertheless, there exists a shared difficulty between seizure prediction and forecasting algorithms: the heterogeneity in epilepsies and seizures. Even with patient-tailored algorithms, as with our methodology, this heterogeneity of results in different patients is evident. Figure 7 shows the Firing Power curve from the SNN ensemble forecasting model for two testing seizures of patient 94402 in (a) and of patient 58602 in (b). There is a clear difference in the models’ performance, as patient 94402’s model overly classifies samples as high-risk, and the opposite happens for patient 58602. There may even be a sizable difference between seizures from the same patient, as shown in Fig. 7c). This figure shows two seizures from patient 30802 using the SNN ensemble forecasting model. On one seizure, the algorithm mainly classifies samples as high-risk; on the other, primarily as low-risk.

### Limitations

This study has some limitations that we should mention. The most significant limiting factor for this study is the database we used. EPILEPSIAE is a presurgical monitoring database with only a few days of data. The recordings belong to patients suffering from medication withdrawal and sleep deprivation to forcefully trigger seizures and analyze them. This procedure results in a more active brain state and a higher number of seizures than that characteristic of everyday life. Using ultra-long-term databases with recordings from several days to months of daily life would help assess the algorithm’s performance more realistically.

Moreover, in this work, we use fixed thresholds for moderate and high-risk states. A patient-specific threshold definition algorithm based on the number of seizures in each state or percentage of time in a high-risk state could significantly improve the model’s performance, as it would adapt and learn from the patient’s seizure frequency. However, the EPILEPSIAE database data, with insufficient seizures and interictal time per patient, does not allow this personalized approach to threshold definition.

## Conclusion

With this study, we aimed to develop methodologies capable of forecasting epileptic seizures and explore possible improvements compared to seizure prediction. To this end, we developed three patient-tailored models with different classifiers based on the most common framework in the literature for each approach.

Looking at the state-of-the-art of seizure prediction and forecasting, we can see that our results were overall poor. The best-performing methodology for prediction used a Logistic Regression classifier and achieved an SS of 0.13±0.26, FPR/h of 0.36±0.40, and IoC of 12.5%. For forecasting, the best-performing model also used the Logistic Regression for classification and achieved 0.28±0.37 for SS, 0.13±0.14 for TiW, 0.19±0.11 for BS, 0.01±0.15 for BSS, and 50% for the IoC. These poor results may be because we used a database with presurgical data and patients monitored with scalp EEG. Other studies, with better performances, use ultra-long-term databases acquired with invasive or sub-scalp EEG during several days or months of regular daily activities.

Comparisons between the results generated by the seizure prediction and forecasting methodologies show a solid conclusion. Looking at the problem through the lens of forecasting produces more optimistic results than seizure prediction. The average SS for the methodologies increased up to 146%, and the number of statistically validated patients likewise showed an improvement of up to 300%. The probabilistic approach of seizure forecasting entirely eliminates the presence of alarms that may even be correctly triggered by brain activity but are rendered useless as standard brain mechanisms terminate the seizure it detected. Allowing patients, caregivers, and clinicians to assess the probability of a seizure and decide accordingly what action to take may be more straightforward and stress-free than often triggering wrong warnings.

It is essential that, even though the conclusions regarding the comparison between the different fields can be extrapolated, the actual performance results should only be viewed as proof of concept, as the study was based on presurgical monitoring data. Furthermore, this work did not consider the existence of other Concept Drifts, such as circadian rhythms or the sleep-wake cycle.

In future work, besides dealing with the limitations we discussed, the developed methodology should have a periodical retraining of the classifier, using the data of all the previous seizures (not just the first three) to assess the risk of the next one, as would happen in a real-life seizure forecasting system. Additionally, the algorithm should be applied to real-life data from an ultra-long-term database, like the one used in the significant first-in-man study published by Cook et al.^26^. Such data would contribute to advancing the wider clinical acceptability of these algorithms and facilitate the evaluation and improvement of current methodologies. Including naturally occurring seizures from ultra-long-term datasets would provide a realistic and comprehensive foundation for research, offering valuable insights into the challenges and opportunities in seizure forecasting.

### Supplementary Information

Supplementary Information.

## Data Availability

This study uses data from the EPILEPSIAE database. The data was provided under license for this study by the EPILEPSIAE Consortium. Therefore, it is not publicly available. However, it can be made available from the corresponding author upon reasonable request and with permission from the EPILEPSIAE Consortium. All the code used in this study is available at https://github.com/GoncaloSantosCosta/Comparison-Between-Epileptic-Seizure-Prediction-and-Forecasting.
