# Supplementary material for: Comparison between epileptic seizure prediction and forecasting based on machine learning
Source: Sci Rep. 2024 Mar 7;14:5653. doi: 10.1038/s41598-024-56019-z (PMC10920642; doi:10.1038/s41598-024-56019-z)
Supplement: Supplementary file 1 — Supplementary Information. [file 41598_2024_56019_MOESM1_ESM.pdf]

Supplementary material for:

Comparison Between Epileptic Seizure Prediction and Forecasting Based on Machine Learning

Gonçalo Costa, César Teixeira, Mauro F. Pinto

1 Patient and seizure metadata

Table S1 contains information on each patient information (gender, age, and number of seizures) and their seizures (seizure classification, seizure activity pattern, state of vigilance at seizure onset, and recording time). The data was obtained from the EPILEPSIAE database[1], and every patient suffers from Temporal Lobe Epilepsy.

Table S1: Information for the 40 studied patients.

Patient ID	Age	Sex	Number of seizures (train/test)	Seizure classification	Seizure activity pattern	Vigilance at seizure onset	Recording duration (h)
402	55	f	3	FOIA, FBTC, FOIA	t, t, t	A, A, A	103.81
			2	FBTC, FOIA	t, t	A, A	29.66
8902	67	f	3	UC, FOIA, FOIA	a, b, a	A, A, A	133.91
			2	FOIA, FOIA	m, a	A, A	22.5
11002	41	m	3	UC, FOIA, FOIA	?, s, a	A, R, A	97.16
			1	FOIA	t	A	11.7
16202	46	f	3	UC, FBTC, UC	r, ?, r	A, A, A	201.32
			4	FOIA, FOIA, FOIA, FOIA	r, r, ?, r	A, A, A, A	34.45
21902	47	m	3	UC, FOIA, FOIA	t, t, t	A, A, A	67.08
			1	FOIA	b	R	9.76
23902	36	m	3	FOA, FOA, FOA	t, t, t	A, A, A	70.74
			2	FOA, FOA	d, t	A, A	33.95
26102	65	m	3	FOIA, FOIA, FOIA	m, t, t	A, A, A	60.65
			1	FOIA	t	A	22.58
30802	28	m	3	FOA, FOA, FOA	t ,t ,t	R, A, 2	87.57
			5	FOA, FOA, FOA, FOA, FOA	t, t, t, t, t	A, A, R, 2, 2	61.71
32702	62	f	3	FOIA, FOIA, FOIA	t ,t ,t	A, A, A	117.38
			2	FOIA, FOIA	r, a	A, A	20.49
45402	41	f	3	FOIA, FOIA, FOA	t, t, t	A, A, A	71.98
			1	FOIA	t	A	22.31
46702	15	f	3	FOA, FOIA, FOIA	a, a, t	A, 2, A	47.46
			2	FBTC, FOIA	b, t	2, A	12.6
50802	43	m	3	FOIA, UC, UC	t, t, t	A, 2, 2	165.93
			2	FOIA, FBTC	t, t	2, A	35.6
52302	61	f	3	UC, FOA, UC	?, ?, d	A, A, 1	76.45
			1	UC	t	A	6.85
53402	39	m	3	FOA, FOA, FOA	?, ?, ?	A, 2, A	70.31
			1	FOIA	t	A	13.73
55202	17	f	3	FOIA, FOIA, FOA	t, d, t	A, A, A	47.05
			5	UC, UC, FOA, UC, FOIA	t, t, t, r, r	A, A, A, A, A	65.37
56402	47	m	3	UC, UC, UC	t, ?, ?	A, A, A	184.22
			1	FBTC	a	A	20.25
58602	32	m	3	FOIA, FOIA, FOIA	r, t, t	A, R, A	96.94
			3	FOIA, FOIA, FOIA	r, r, t	A, A, 2	23.34
59102	47	m	3	FOA, FOIA, FOIA	?, t, t	A, A, A	65.83
			2	FOIA, FOA	t, t	A, A	82.22
60002	55	m	3	FOIA, FOIA, FOIA	d, c, t	1, A, A	208.11
			3	UC, FOIA, FOIA	t, d, d	R, R, 1	152.4
64702	51	m	3	FOA, FBTC, FBTC	?, m, t	A, A, A	75.91
			2	FBTC, FBTC	t, t	A, 2	31.59
75202	13	m	3	FOA, FOA, UC	t, t, t	2, 2, A	100.94
			4	FOA, FOA, FOA, FOA	t, t, ?, t	A, A, A, A	52.63

Table S1 – *Continued from previous page*

Patient ID	Age	Sex	Number of seizures (train/test)	Seizure classification	Seizure activity pattern	Vigilance at seizure onset	Recording duration (h)
80702	22	f	3	FOIA, FOIA, UC	b, b, ?	A, A, A	49.4
			3	FOIA, FBTC, FOIA	c, c, c	A, A, A	29.55
85202	54	f	3	FOIA, FOIA, UC	m, c, m	2, A, A	53.49
			2	UC, UC	m, m	A, A	20.42
93402	67	m	3	FBTC, FOIA, FOIA	t, t, t	2, 2, 2	98.0
			2	UC, UC	t, t	2, 2	54.07
93902	50	m	3	FOA, FOIA, FBTC	t, t, d	A, A, 2	370.83
			3	FOIA, FOIA, UC	d, d, d	A, 2, A	20.29
94402	37	f	3	FOA, UC, FOIA	?, d, b	A, A, A	120.23
			4	UC, FOA, UC, FOA	t, ?, b, ?	2, A ,2, A	30.37
95202	50	f	3	FBTC, FOIA, FOIA	b, b, b	2, 2, 2	57.6
			4	FOIA, UC, FOIA, UC	m, b, b, t	2, 2, 2, 2	89.53
96002	58	m	3	FOIA, FOIA, FOIA	t, t, t	A, A, A	48.4
			4	FOIA, UC ,FOIA, FOIA	d ,a ,t ,a	A, A, A, A	82.2
98102	36	m	3	FOA, UC, UC	?, ?, ?	A, A, A	108.61
			2	UC, FBTC	?, ?	A, A	45.68
98202	39	m	3	FOIA, FOIA, FOIA	t, a, t	A, A, A	111.33
			5	FBTC, FOIA, FOIA, FOIA, UC	t, t, t, t, t	A, A, A, A, A	49.88
101702	52	m	3	FOIA, FOIA, FOIA	t, t, t	A, A, A	28.41
			2	FOIA, FOIA	r, r	2, A	23.83
102202	17	m	3	FOA, UC, FOIA	b, ?, t	2, A, 2	57.45
			4	UC, FOA, FOIA, UC	?, t, t, t	A, A, 2, A	51.41
104602	17	f	3	FOIA, FBTC, FBTC	t, a, t	A, 2, 2	87.87
			2	FBTC, UC	t, d	2, 2	15.25
109502	50	m	3	FOIA, FOIA, UC	t, t, t	A, A, A	76.8
			1	UC	t	A	41.94
110602	56	m	3	FOIA, FOIA, FOIA	t, t, t	A, A, A	89.63
			2	FOIA, FOA	t, t	A, A	25.92
112802	52	m	3	UC, FOIA, UC	t, t, t	A, A, A	71.58
			3	FOIA, FOIA, UC	t, t, t	A, A, A	111.5
113902	29	f	3	UC, FOIA, FOIA	t, d, t	A, A, 2	61.98
			3	FOIA, UC, FOIA	t, t, t	A, 2, A	22.73
114702	22	f	3	FOIA, FOIA, UC	t, t, t	A, A, A	68.39
			5	FOIA, FOIA, FOIA, FOIA, FOIA	t, d, t, d, t	A, A, A, A, A	34.04
114902	16	f	3	FOA, FOIA, FOIA	s, b, s	A, A, A	26.55
			4	FBTC, UC, FOIA, FOIA	t, r, a, t	2, A, A, A	50.66
123902	25	f	3	FBTC, FBTC, FOIA	t, t, t	2, 2, R	152.11
			2	FOIA, FOA	t, t	A, A	30.15

Gender: female (f), male (m); Seizure classification: unclassified (UC), Focal Onset Aware (FOA), Focal Onset Impaired (FOIA), Focal to Bilateral Tonic-Clonic (FBTC); Seizure activity pattern: unclear (?), rhythmic sharp waves (s), alpha waves (a), rhythmic delta waves (d), rhythmic theta waves (t), rhythmic beta waves (b), repetitive spiking (r), cessation of interictal activity (c), amplitude depression (m); Vigilance state: awake (A), REM sleep stage (R), Non-REM sleep stage I (1), Non-REM sleep stage II (2).

2 Feature details

Here, we present a detailed description of the extracted features. Linear features are mathematical measures using amplitude and phase/frequency information to extract linear aspects from signals. These features assume that the EEG signal is quasi-stationary within each time window.

Statistical Moments

Statistical moments can characterize to some extent the amplitude distribution of the EEG time series. The first four statistical moments are the mean (the central tendency of the samples' amplitude), the variance (the dispersion of the samples' amplitude around its mean), the skewness (the degree of asymmetries of the amplitude distribution), and the kurtosis (the relative flatness or peakedness of the amplitude distribution). Comparing the preictal period with the interictal one shows significant changes to these statistical measures. Specifically, the preictal stage is connected to an increase in kurtosis and a decrease in variance[2–8].

Hjörth Parameters

Hjörth parameters detect an increase of energy in the brain caused by an intensification of brain activity. These parameters are activity, mobility, and complexity. Hjörth activity is measured through amplitude variance, and the variance of the time function can be interpreted as the surface of the power spectrum in the frequency domain. Hjörth mobility is an estimate of the mean frequency obtained by quantifying the variance of a time-series' slopes normalized by the variance of its amplitude distribution. Hjörth complexity is an estimate of the bandwidth and is defined as the variance of the rate of slope changes with an ideal sinusoid as a reference. Studies show that during the preictal period, there is a substantial rise in the mobility and complexity of the EEG[2–6].

Decorrelation Time

Decorrelation time represents the first zero-crossing of the autocorrelation function. It is a measure that detects repeating patterns or identifies the fundamental frequency underlying its harmonic frequencies. Additionally, decorrelation time provides information about the typical time scale of data variability and can be used to measure signal stochasticity. A value of 0 indicates that a signal is entirely stochastic or white noise. Decreases in decorrelation time have been observed before seizures, indicating its potential for detecting preictal periods[2–4,6].

Relative Spectral Power

Features in the frequency domain can be used to capture shifts from low to high frequencies. The EEG signal is decomposed into frequency bands (delta, theta, alpha, beta, and gamma), and their spectral power is computed. These are the most widely used features and can be determined using the Power Spectral Density (PSD) of the time series within a time window. One of the ways to calculate PSD is to perform the Fast Fourier Transform (FFT) on the time series and average the squared coefficients of the frequency range of interest. This calculation assumes the signal in each window is long enough to capture the low-frequency activity of the brain but short enough to be considered quasi-stationary. Several studies suggest a transfer of PSD from lower to higher frequencies before seizure onset[2–4,6,9].

Spectral Edge Frequency and Power

Typically, the spectral power of the EEG signal is distributed between 0 Hz and 40 Hz. The Spectral Edge Frequency (SEF) and the Spectral Edge Power (SEP) quantify the power distribution within this frequency range. The SEF is the threshold frequency under which x percentage of the signal's full spectral power is encompassed. The SEP is the value of power existing under this defined threshold. For the mentioned 0-40 Hz frequency band characteristic of EEG, the value of $x = 50\%$ is widely used. Therefore, the SEF may be able to capture the shift from lower to higher frequencies in the preictal period that many studies suggest[2,5].

Wavelet Coefficients Energy

The Discrete Wavelet Transform (DWT) is a time-frequency domain transform that decomposes the signal in different resolution levels according to different frequency ranges. It can be an alternative to the FFT. Wavelet analysis decomposes a signal into time-variant frequency components. It provides a high-frequency resolution for lower frequencies and a high time resolution for higher ones, capturing sudden changes and minor details in the signal. By computing the energy of the signal components generated by the decomposition, it is possible to measure the energy in different frequency ranges[2,4,6,10–14].

3 Results obtained for all approaches

Tables S2, S3, and S4 contain the grid-search results for the optimal parameters and the performance results for the Logistic Regression, the 15 SVM ensemble, and the 15 SNN ensemble, respectively.

Table S2: Grid-search and testing results for the Logistic Regression model. SOP stands for Seizure Occurrence Period, SS for Sensitivity, FPR/h for False Positive Rate per Hour, IoC for Improvement over Chance, TiW for Time in Warning, BS for Brier Score, and BSS for Brier Skill Score.

		Grid-Search		Prediction			Forecasting					
**Patient**	**Tested seizures**	**SOP (mins)**	**k-features**	**SS**	**FPR/h**	**IoC SS**	**SS**	**TiW**	**BS**	**BSS**	**IoC SS**	**IoC BS**
402	2	50	20	0.00	0.00		0.00	0.00	0.17	-0.05		
8902	2	25	7	1.00	0.10	X	1.00	0.10	0.08	0.21	X	X
11002	1	20	7	0.00	0.71		0.00	0.16	0.28	0.00		X
16202	4	20	30	0.00	0.03		0.00	0.01	0.07	-0.08		
21902	1	40	10	0.00	0.00		0.00	0.00	0.10	-0.15		
23902	2	45	15	0.50	1.09		1.00	0.25	0.31	0.13	X	X
26102	1	50	30	0.00	0.00		0.00	0.00	0.15	-0.02		
30802	5	50	5	0.20	0.37		0.80	0.41	0.30	-0.23	X	
32702	2	20	7	0.50	0.05	X	0.50	0.04	0.07	0.22	X	X
45402	1	25	20	0.00	0.55		0.00	0.09	0.23	0.01		X
46702	2	30	20	0.00	0.00		0.00	0.00	0.20	0.00		
50802	2	20	10	0.00	0.26		0.00	0.05	0.07	0.23		X
52302	1	45	30	0.00	0.94		0.00	0.43	0.51	-0.06		
53402	1	40	20	0.00	0.27		1.00	0.14	0.17	0.16	X	X
55202	5	45	3	0.20	0.52		0.80	0.29	0.28	-0.01	X	
56402	1	20	10	0.00	0.51		0.00	0.11	0.19	0.00		
58602	3	20	3	0.00	0.52		0.00	0.14	0.25	-0.04		
59102	2	35	30	0.50	0.99		1.00	0.40	0.37	-0.14	X	
60002	3	35	7	0.00	0.05		0.00	0.06	0.18	-0.09		
64702	2	40	3	0.00	0.52		0.50	0.15	0.19	0.19	X	X
75202	4	25	30	0.00	0.04		0.00	0.01	0.08	-0.07		
80702	3	40	30	0.33	0.27	X	0.33	0.09	0.24	0.09	X	X
85202	2	20	20	0.00	0.11		0.00	0.03	0.08	-0.03		
93402	2	20	3	1.00	0.46	X	1.00	0.33	0.31	-0.60	X	
93902	3	45	5	0.00	0.12		0.33	0.04	0.15	0.16	X	X
94402	4	45	30	0.00	0.71		0.25	0.20	0.31	0.00		
95202	4	25	15	0.00	0.34		0.25	0.08	0.13	0.12	X	X
96002	4	25	3	0.25	0.52		0.25	0.18	0.23	0.09		X
98102	2	25	20	0.00	0.12		0.50	0.03	0.07	0.16	X	X
98202	5	25	30	0.00	0.02		0.00	0.01	0.15	0.02		X
101702	2	50	30	0.00	0.66		0.50	0.32	0.27	0.02		X
102202	4	50	3	0.00	0.04		0.00	0.02	0.09	0.10		X
104602	2	35	30	0.00	0.37		0.00	0.10	0.20	0.09		X
109502	1	30	20	0.00	1.95		0.00	0.43	0.44	-0.02		
110602	2	40	10	0.50	0.31	X	0.50	0.17	0.19	0.10	X	X
112802	3	20	3	0.33	0.68		0.67	0.27	0.28	-0.19	X	
113902	3	45	30	0.00	0.05		0.00	0.04	0.17	0.01		X
114702	5	35	30	0.00	0.00		0.00	0.00	0.13	-0.06		
114902	4	25	10	0.00	0.00		0.00	0.00	0.05	0.17		X
123902	2	25	3	0.00	0.00		0.00	0.00	0.03	-0.08		

Table S3: Grid-search and testing results for the SVM ensemble model. SOP stands for Seizure Occurrence Period, SS for Sensitivity, FPR/h for False Positive Rate per Hour, IoC for Improvement over Chance, TiW for Time in Warning, BS for Brier Score, and BSS for Brier Skill Score.

		**Grid-Search**			**Prediction**			**Forecasting**					
**Patient**	**Tested seizures**	**SOP (mins)**	**k-features**	**C-value**	**SS**	**FPR/h**	**IoC SS**	**SS**	**TiW**	**BS**	**BSS**	**IoC SS**	**IoC BS**
402	2	50	30	2^{-2}	0.00	0.00		0.00	0.00	0.13	-0.50		
8902	2	20	20	2^{-8}	0.50	0.20	X	1.00	0.15	0.11	0.00	X	
11002	1	20	15	2^{-10}	0.00	2.69		1.00	0.46	0.45	-0.01	X	
16202	4	20	15	2^{0}	0.00	0.06		0.00	0.02	0.08	-0.01		
21902	1	35	7	2^{-2}	0.00	0.00		0.00	0.00	0.14	-0.16		
23902	2	45	10	2^{-8}	0.00	1.38		0.50	0.31	0.37	0.08		X
26102	1	50	30	2^{2}	0.00	0.10		0.00	0.04	0.15	0.00		X
30802	5	50	15	2^{-10}	0.60	0.38	X	0.80	0.44	0.36	-0.25	X	
32702	2	20	30	2^{8}	0.00	0.11		0.00	0.01	0.07	0.03		X
45402	1	20	15	2^{2}	0.00	0.85		0.00	0.16	0.24	-0.05		
46702	2	50	30	2^{4}	0.00	0.00		0.00	0.00	0.19	-0.04		
50802	2	20	30	2^{-8}	0.00	0.16		0.00	0.04	0.06	0.21		X
52302	1	50	3	2^{6}	0.00	1.05		0.00	0.19	0.31	-0.15		
53402	1	40	3	2^{-10}	0.00	0.27		1.00	0.16	0.23	0.11	X	X
55202	5	20	20	2^{-10}	0.20	1.06		0.20	0.26	0.28	-0.09		
56402	1	20	15	2^{-10}	0.00	3.45		0.00	0.53	0.48	-0.02		
58602	3	20	3	2^{-10}	0.00	0.00		0.00	0.00	0.06	-0.14		
59102	2	50	15	2^{-4}	0.00	0.59		0.00	0.27	0.31	-0.13		
60002	3	25	10	2^{8}	0.33	0.32		0.33	0.28	0.31	-0.04		
64702	2	35	3	2^{-10}	0.00	1.40		0.50	0.29	0.28	0.23		X
75202	4	30	30	2^{-10}	0.00	0.04		0.00	0.02	0.09	-0.13		
80702	3	45	15	2^{-10}	0.67	0.48	X	0.67	0.21	0.27	0.11	X	X
85202	2	20	20	2^{-10}	0.50	1.21	X	1.00	0.27	0.29	0.05	X	X
93402	2	20	5	2^{-8}	0.50	1.20		1.00	0.30	0.31	-0.12	X	
93902	3	35	3	2^{-10}	0.00	0.06		0.00	0.01	0.13	0.04		X
94402	4	20	20	2^{-4}	0.00	1.54		0.00	0.29	0.33	0.02		X
95202	4	25	30	2^{-10}	0.25	0.62		0.25	0.19	0.20	0.10		X
96002	4	45	20	2^{8}	0.00	0.94		0.25	0.42	0.46	-0.05		
98102	2	25	15	2^{-10}	0.50	0.17	X	0.50	0.05	0.10	0.09	X	X
98202	5	20	10	2^{8}	0.00	1.55		0.00	0.23	0.27	0.18		X
101702	2	20	15	2^{6}	0.00	0.87		0.50	0.19	0.22	0.04		X
102202	4	50	5	2^{-6}	0.00	0.36		0.50	0.18	0.28	0.00	X	X
104602	2	25	3	2^{-10}	0.50	2.67		1.00	0.41	0.38	0.08	X	X
109502	1	50	5	2^{-4}	0.00	0.29		0.00	0.15	0.23	-0.05		
110602	2	25	30	2^{-10}	0.00	0.45		0.50	0.16	0.20	0.04	X	X
112802	3	35	3	2^{4}	0.00	2.22		0.67	0.65	0.60	-0.12		
113902	3	20	7	2^{-10}	0.33	0.21	X	0.33	0.05	0.17	0.03	X	X
114702	5	35	10	2^{-2}	0.00	0.00		0.00	0.00	0.13	-0.02		
114902	4	20	3	2^{-10}	0.25	0.20	X	0.25	0.06	0.08	0.24	X	X
123902	2	20	3	2^{-2}	0.00	0.00		0.00	0.00	0.04	-0.09		

Table S4: Grid-search and testing results for the SNN ensemble model. SOP stands for Seizure Occurrence Period, SS for Sensitivity, FPR/h for False Positive Rate per Hour, IoC for Improvement over Chance, TiW for Time in Warning, BS for Brier Score, and BSS for Brier Skill Score.

		**Grid-Search**	**Prediction**			**Forecasting**					
**Patient**	**Tested seizures**	**SOP (mins)**	**SS**	**FPR/h**	**IoC SS**	**SS**	**TiW**	**BS**	**BSS**	**IoC SS**	**IoC BS**
402	2	20	0,00	0,11		0,00	0,03	0,12	-0,23		
8902	2	20	0,50	0,10	X	1,00	0,06	0,07	0,23	X	X
11002	1	20	0,00	0,42		1,00	0,11	0,16	0,08	X	X
16202	4	20	0,00	0,10		0,00	0,03	0,09	-0,09		
21902	1	35	0,00	0,67		0,00	0,23	0,25	-0,14		
23902	2	20	0,00	0,93		0,00	0,25	0,33	-0,09		
26102	1	20	0,00	0,09		0,00	0,01	0,10	0,03		X
30802	5	25	0,60	0,56	X	0,80	0,37	0,29	-0,37	X	
32702	2	35	0,00	0,00		0,00	0,00	0,12	-0,38		
45402	1	20	0,00	1,69		0,00	0,32	0,34	-0,04		
46702	2	35	0,00	0,00		0,00	0,00	0,10	-0,03		
50802	2	25	0,00	0,09		0,00	0,02	0,06	0,10		X
52302	1	20	0,00	0,63		0,00	0,11	0,19	0,00		
53402	1	40	0,00	0,27		0,00	0,10	0,17	0,13		X
55202	5	25	0,20	1,33		0,80	0,44	0,37	-0,11		
56402	1	25	0,00	1,74		0,00	0,37	0,41	-0,03		
58602	3	45	0,00	0,00		0,00	0,00	0,12	-0,25		
59102	2	35	0,50	1,27		1,00	0,38	0,37	0,01	X	X
60002	3	20	0,33	0,22	X	0,33	0,16	0,23	0,00		
64702	2	25	0,00	0,27		0,00	0,06	0,12	0,19		X
75202	4	20	0,50	0,24	X	0,50	0,08	0,13	-0,08	X	
80702	3	20	0,33	0,00	X	0,33	0,00	0,06	0,11	X	X
85202	2	20	0,00	0,44		0,00	0,12	0,21	-0,06		
93402	2	20	0,00	0,19		0,00	0,12	0,22	-0,57		
93902	3	20	0,00	0,24		0,00	0,08	0,12	-0,08		
94402	4	20	0,50	3,37	X	0,50	0,50	0,43	0,04		X
95202	4	20	0,25	0,30	X	0,25	0,10	0,13	-0,01		
96002	4	45	0,25	0,31		0,25	0,15	0,25	-0,08		
98102	2	20	0,00	0,05		0,00	0,01	0,06	0,12		X
98202	5	25	0,00	0,25		0,00	0,04	0,16	0,00		X
101702	2	20	0,00	1,15		0,50	0,26	0,27	0,03		X
102202	4	30	0,00	0,11		0,25	0,02	0,09	0,13	X	X
104602	2	30	0,50	0,47	X	0,50	0,11	0,19	0,15	X	X
109502	1	25	0,00	0,05		0,00	0,01	0,10	-0,03		
110602	2	20	0,00	0,70		0,00	0,17	0,19	0,04		X
112802	3	25	0,33	0,69		0,67	0,40	0,39	-0,52	X	
113902	3	20	0,00	0,05		0,00	0,00	0,07	0,13		X
114702	5	25	0,00	0,14		0,00	0,02	0,10	0,01		X
114902	4	30	0,00	0,13		0,25	0,05	0,10	0,22	X	X
123902	2	25	0,00	0,00		0,00	0,00	0,03	-0,07		

References

1. Klatt J, Feldwisch-Drentrup H, Ihle M, Navarro V, Neufang M, Teixeira C, et al. The EPILEPSIAE database: An extensive electroencephalography database of epilepsy patients. Epilepsia. 2012 9;53(9):1669-76.
2. Teixeira CA, Direito B, Bandarabadi M, Le Van Quyen M, Valderrama M, Schelter B, et al. Epileptic seizure predictors based on computational intelligence techniques: A comparative study with 278 patients. Computer methods and programs in biomedicine. 2014;114(3):324-36.
3. Mormann F, Kreuz T, Rieke C, Andrzejak RG, Kraskov A, David P, et al. On the predictability of epileptic seizures. Clinical neurophysiology. 2005;116(3):569-87.
4. Rasekhi J, Mollaei MRK, Bandarabadi M, Teixeira CA, Dourado A. Preprocessing effects of 22 linear univariate features on the performance of seizure prediction methods. Journal of neuroscience methods. 2013;217(1-2):9-16.
5. Mormann F, Andrzejak RG, Elger CE, Lehnertz K. Seizure prediction: the long and winding road. Brain. 2007;130(2):314-33.
6. Direito B, Teixeira CA, Sales F, Castelo-Branco M, Dourado A. A realistic seizure prediction study based on multiclass SVM. International journal of neural systems. 2017;27(03):1750006.
7. Siddiqui MK, Islam MZ. Data mining approach in seizure detection. In: 2016 IEEE Region 10 Conference (TENCON). IEEE; 2016. p. 3579-83.
8. Siddiqui MK, Morales-Menendez R, Huang X, Hussain N. A review of epileptic seizure detection using machine learning classifiers. Brain informatics. 2020;7(1):1-18.
9. Bandarabadi M, Teixeira CA, Rasekhi J, Dourado A. Epileptic seizure prediction using relative spectral power features. Clinical Neurophysiology. 2015;126(2):237-48.
10. Kuhlmann L, Karoly P, Freestone DR, Brinkmann BH, Temko A, Barachant A, et al. Epilepsyecosystem. org: crowdsourcing reproducible seizure prediction with long-term human intracranial EEG. Brain. 2018;141(9):2619-30.
11. Karoly PJ, Ung H, Grayden DB, Kuhlmann L, Leyde K, Cook MJ, et al. The circadian profile of epilepsy improves seizure forecasting. Brain. 2017;140(8):2169-82.
12. Rasekhi J, Mollaei MRK, Bandarabadi M, Teixeira CA, Dourado A. Epileptic seizure prediction based on ratio and differential linear univariate features. Journal of medical signals and sensors. 2015;5(1):1.
13. Moghim N, Corne DW. Predicting epileptic seizures in advance. PloS one. 2014;9(6):e99334.
14. Cook MJ, O'Brien TJ, Berkovic SF, Murphy M, Morokoff A, Fabinyi G, et al. Prediction of seizure likelihood with a long-term, implanted seizure advisory system in patients with drug-resistant epilepsy: a first-in-man study. The Lancet Neurology. 2013;12(6):563-71.
